# Acute toxic effects of endosulfan on cellular Stress, genotoxicity, and histopathological alterations in Milkfish (*Chanos chanos*) under freshwater conditions

**DOI:** 10.3389/fimmu.2026.1887110

**Published:** 2026-09-30

**Authors:** Neeraj Kumar, K. Ambasankar, Dilip Kumar Singh, Kotha Sammi Reddy

**Affiliations:** 1 ICAR-National Institute of Abiotic Stress Management, Baramati, Pune, India; 2 ICAR-Central Institute of Brackishwater Aquaculture, Chennai, India; 3 ICAR-Central Institute of Fisheries Education, Kolkata Center, Kolkata, India

**Keywords:** antioxidative status, Chanos chanos, endosulfan, genotoxicity, histopathology, metabolic enzymes

## Abstract

**Background:**

The present study aimed to evaluate the acute toxicity of endosulfan (99% purity; α:β ratio of 7:3) in milkfish (*Chanos chanos*) under freshwater acclimated conditions over a 96-hour exposure period. Milkfish (*C. chanos*) is a commercially important tropical species widely cultured across freshwater, brackish, marine, and hypersaline environments.

**Method:**

An experiment was conducted in Milkfish (*C. chanos*) acclimated under freshwater condition to determine the lethal concentration (LC_50_) of endosulfan at 96 hrs using probit analysis. The fish were exposed to graded concentrations of endosulfan (25.5, 26.5, 27.5, 28.5, and 29.5 µg L^-^¹) for 96 hours. Toxicological effects were evaluated through a suite of biochemical, immunological, neurophysiological, cytogenetic, and histopathological biomarkers. Antioxidative enzyme activities, including superoxide dismutase, glutathione-S-transferase, and catalase, were assessed alongside immunological parameters such as blood glucose, phagocytic activity (measured via nitroblue tetrazolium reduction), and serum cortisol levels. Neurotoxicity was evaluated through acetylcholinesterase activity, while chromosomal aberrations and histopathological alterations in the liver and gills were examined to determine cellular and tissue-level damage.

**Results:**

The 96-hour LC_50_ value (95% confidence limits) for *C. chanos* (mean body weight: 110 g) was estimated at 27.47 µg L^-^¹. A significant dose and time dependent alteration was observed across all assessed parameters, including antioxidative enzymes, immune responses, neurotransmitter activity, metabolic functions, and cytogenetic integrity. Pronounced chromosomal aberrations in kidney tissues and severe histopathological lesions in the liver and gills were evident following endosulfan exposure. These physiological and structural impairments were further corroborated by marked behavioral deviations from normal activity patterns in exposed fish.

**Conclusions:**

Collectively, the findings demonstrate that endosulfan exposure induces substantial biochemical, cytogenetic, and histopathological disruptions in *C. chanos*, ultimately leading to metabolic dysregulation and organ damage.

## Introduction

The indiscriminate use of chemicals worldwide has led to widespread environmental pollution, posing significant toxicity risks to non-target organisms (1–3). Consequently, the adverse impacts of anthropogenic activities on aquatic ecosystems have become a major environmental concern (4). Variations in physicochemical parameters, such as water temperature, dissolved oxygen, pH, and salinity, along with biological stressors including food availability and pathogen load, can profoundly influence the health and survival of aquatic organisms. Among chemical contaminants, pesticides are considered one of the most detrimental agents affecting aquatic environments. In particular, organochlorine pesticides are regarded as highly hazardous due to their persistence, resistance to biodegradation, and tendency to bioaccumulate and biomagnify within food chains (5). Endosulfan, a cyclodiene organochlorine insecticide, exists as two stereoisomers: α-endosulfan (64-67%) and β-endosulfan (29-32%). It has been extensively used worldwide for pest control, thereby contributing to increased agricultural productivity. However, residues of endosulfan and its metabolites have been frequently associated with fish mortality in freshwater and estuarine ecosystems (6–8). The persistence of endosulfan in aquatic environments is notable, with a reported half-life ranging from 35 to 150 days (9). It ultimately degrades into major by products such as endosulfan sulfate and endosulfan lactone, which are often more persistent and, in some cases, more toxic than the parent compound (10). The toxic mode of action of endosulfan is primarily attributed to its chlorinated cyclodiene structure, which disrupts the electrophysiological and enzymatic properties of neuronal membranes. Specifically, it alters the kinetics of sodium (Na^+^) and potassium (K^+^) ion transport across membranes and interferes with the function of the inhibitory neurotransmitter gamma-aminobutyric acid (GABA). By blocking GABA gated chloride channels, endosulfan inhibits chloride ion influx into neurons, leading to neuroexcitation (11). This neurotoxic mechanism likely underlies the high sensitivity of fish and other aquatic organisms to endosulfan exposure (12). Notably, concentrations of organochlorine pesticides in marine waters surrounding India have been reported to be significantly higher than in many other regions (13), with substantial residues of endosulfan detected in Indian estuarine systems (14).

Milkfish (*Chanos chanos*) is an important tropical brackishwater species widely cultured in countries such as the Philippines, Indonesia, India, and Taiwan. Global production of milkfish has been estimated at approximately 330,000 tonnes annually (15, 16), with more recent reports indicating an increase to 943,259 tonnes (17). This species occurs naturally and is commercially cultivated across a wide range of environments, including freshwater, brackishwater, marine, and hypersaline lagoons (18). Despite its economic importance, *C. chanos* has been relatively understudied with respect to toxicological responses and thermal tolerance (19–21).

Exposure to environmental contaminants in aquatic ecosystems can enhance the intracellular generation of reactive oxygen species (ROS), leading to oxidative damage in biological systems (22). Various pesticides are known to induce both *in vitro* and *in vivo* production of ROS, including hydrogen peroxide, superoxide anions, and hydroxyl radicals (23). Oxidative stress occurs when there is an imbalance between ROS generation and the antioxidant defense system. These reactive species are neutralized by enzymatic defense mechanisms, including superoxide dismutase (SOD) and catalase (CAT), while organic peroxides are detoxified by glutathione-S-transferase (GST) (24). Alterations in antioxidant enzyme activities have been widely recognized as reliable biomarkers of environmental stress in aquatic organisms (25). Pesticide exposure has also been reported to alter transaminase activities, such as alanine aminotransferase (ALT) and aspartate aminotransferase (AST), which are indicative of liver dysfunction (26) (Eliska et al., 2009) and gill damage (27). Similarly, enzymes such as lactate dehydrogenase (LDH) and malate dehydrogenase (MDH) have been used as sensitive indicators of tissue damage in fish under toxic stress (28, 29). Monitoring changes in these enzymatic activities can therefore provide valuable insights into stress-induced physiological and metabolic disturbances in fish exposed to xenobiotics.

To the best of our knowledge, this study represents the first comprehensive investigation of the acute effects of endosulfan exposure in freshwater-acclimated milkfish (*C. chanos*), focusing on its impact on overall physiological status. Accordingly, the present study aimed to evaluate the effects of 96-hour acute exposure to endosulfan on metabolic, neurophysiological, and antioxidative enzymes, as well as on genotoxicity (chromosomal aberrations) and histopathological alterations in this species.

## Materials and methods

### Ethics statement

NIASM aquaculture Central Wet Laboratory facilities and experimental protocols were approved under the Committee for the Purpose of Control and Supervision of Experiments on Animals (CCSEA) under the number 2190/GO/RReBi/SL/2022/CCSEA. This study also strictly adhered to the ARRIVE (Animal Research: Reporting of *In Vivo* Experiments) guidelines. All methods were performed in accordance with the relevant guidelines and regulations.

### Experimental animals

Juvenile milkfish (*Chanos chanos*) used in the present study were obtained from the Central Institute of Brackishwater Aquaculture (CIBA), Muttukadu Research Centre, Chennai, India. The fish had an average body weight of 110 g and were acclimatized to freshwater conditions for 25 days prior to experimentation. During this period, they were maintained in circular fiberglass tanks (500 L capacity) at a stocking density of 10 fish per tank.

### Experimental conditions

The experimental tanks were continuously aerated throughout the study period. Water temperature was maintained within the range of 26.4-28.8 °C, and salinity was kept at 0 ppt. Fish were fed a formulated diet containing 30% crude protein at a daily ration equivalent to 3% of their body weight until 48 hours prior to the commencement of the experiment. Physicochemical parameters of the water were monitored regularly during the experimental period. Dissolved oxygen and temperature were measured using a dissolved oxygen–temperature meter (Merck, Germany), while pH was determined using a digital pH meter (LABINDIA, Mumbai). Free carbon dioxide was estimated by the titrimetric method following APHA (30). Total hardness was measured using a carbonate hardness test kit (Merck, Germany). Ammonia concentration was determined at 635 nm using the phenate method (30), whereas nitrite and nitrate levels were measured spectrophotometrically at 543 nm according to standard methods described by APHA (30).

### Determination of lethal concentration 50 (LC_50_)

A static, non-renewable acute toxicity bioassay was conducted following standard methods of APHA (30) to determine the 96-hour LC_50_ of endosulfan in milkfish (*Chanos chanos*) (mean body weight: 110 g). The quality and preparation of endosulfan have been described in our previous studies (31–34). Preliminary range-finding tests were performed in triplicate to establish the concentration range for the definitive assay. In these trials, fish were exposed to a series of endosulfan concentrations on a logarithmic scale (1, 5, 7, 10, 15, 20, 25, and 30 µg L^-^¹). Based on the results, the LC_50_ range for *C. chanos* was estimated to lie between 25 and 29 µg L^-^¹ under the given experimental conditions. For the definitive test, five concentrations of endosulfan (25.5, 26.5, 27.5, 28.5, and 29.5 µg L^-^¹) were selected. Each concentration was tested in triplicate using a static, non-renewable system, with 10 fish per tank. No water exchange was performed, and fish were not fed during the exposure period of 96 hr. Cumulative mortality was recorded at 24, 48, 72, and 96 hours. A control group was maintained using the maximum volume of ethanol (vehicle control) employed for the preparation of test solutions. Dead fish were removed immediately upon observation. At the end of the 96-hour exposure period, surviving fish were anesthetized with clove oil (50 mg kg^-^¹) and blood samples were collected. Subsequently, fish were euthanized by decapitation and dissected under aseptic conditions to isolate gill, liver, and brain tissues for biochemical and hematological analyses. The mortality data were analyzed using probit analysis to estimate LC_50_ values, employing the computer program Basic LD_50_ version 1.1 (35).

### Tissue homogenate preparation

Fish were anesthetized with clove oil (50 mg kg^-1^) in emersion method technique before collection of different tissue such as gill, liver, and brain from both exposed and control groups were carefully dissected and weighed. The tissues were homogenized in ice-cold 0.25 M sucrose solution using a Teflon-coated mechanical tissue homogenizer (MICCRA D-9, Digitronic, Germany). Throughout the homogenization process, samples were maintained on ice to prevent thermal denaturation of enzymes and ensure analytical accuracy. The homogenates were centrifuged at 6000 rpm for 20 minutes at 4 °C using a refrigerated centrifuge. The resulting supernatants were collected and stored at -20 °C until further biochemical analysis. A 5% (w/v) tissue homogenate was prepared from gill, liver, and brain samples for enzymatic assays.

### Biochemical assays

#### Antioxidant enzymes

##### Superoxide dismutase (SOD)

Superoxide dismutase (SOD; EC 1.15.1.1) activity was determined following the method of Misra and Fridovich (36), based on the inhibition of epinephrine auto-oxidation to adrenochrome. The assay mixture comprised 50 µl of tissue homogenate, 1.5 ml of phosphate buffer, and 0.5 ml of freshly prepared epinephrine. The change in absorbance was recorded immediately at 480 nm for 3 minutes using a Shimadzu UV–visible spectrophotometer. Enzyme activity was expressed as units per mg protein.

##### Catalase (CAT)

Catalase (CAT; EC 1.11.1.6) activity was measured according to the method of Takahara et al. (37). The reaction mixture contained 2.45 ml of phosphate buffer (50 mM; pH 7.0), 50 µl of tissue homogenate, and 1 ml of freshly prepared hydrogen peroxide. The decomposition of hydrogen peroxide was monitored as a decrease in absorbance at 240 nm over a period of 3 minutes. Catalase activity was expressed as units per mg protein.

##### Gluthathione-S-transferase (GST)

Glutathione S-transferase (GST; EC 2.5.1.18) activity was assayed spectrophotometrically following Habig et al. (38), using 1-chloro-2,4-dinitrobenzene (CDNB) as the substrate. The assay is based on the conjugation of CDNB with reduced glutathione, resulting in the formation of a thioether adduct. The increase in absorbance was monitored at 340 nm against an appropriate blank. GST activity was expressed as units per mg protein.

##### Acetylcholine esterase (AChE)

Acetylcholinesterase (AChE; EC 3.1.1.7) activity was determined spectrophotometrically following the method of Hestrin, as modified by Augustinsson (39). The assay was based on the hydrolysis of acetylcholine iodide, using dithiobisnitrobenzoic acid (DTNB) as the chromogenic reagent. The rate of increase in absorbance was monitored at 540 nm against a reagent blank, and enzyme activity was expressed as units per mg protein.

##### Metabolic enzymes

Lactate dehydrogenase (LDH; EC 1.1.1.27) activity was measured using 0.1 M phosphate buffer (pH 7.5) containing 0.2 mM NADH. The reaction was initiated by the addition of sodium pyruvate (0.2 mM), and the decrease in absorbance due to NADH oxidation was recorded at 340 nm (40). Malate dehydrogenase (MDH; EC 1.1.1.37) activity was assayed under similar conditions, with oxaloacetate (1 mg mL^-^¹ in chilled triple-distilled water) serving as the substrate (41).

Aspartate aminotransferase (AST; EC 2.6.1.1) and alanine aminotransferase (ALT; EC 2.6.1.2) activities were determined by quantifying the formation of oxaloacetate and pyruvate, respectively, following incubation of the reaction mixture at 37 °C for 60 minutes (42).

Glucose-6-phosphate dehydrogenase (G6PDH; EC 1.1.1.49) activity was estimated according to the method of De Moss (43). The reaction mixture consisted of 1.5 ml Tris buffer (0.1 M, pH 7.8), 0.2 ml NADP (2.7 mM), 0.1 ml tissue homogenate, 1.05 ml distilled water, and 0.1 ml glucose-6-phosphate (0.02 M). The formation of NADPH was monitored by measuring the increase in absorbance at 340 nm.

### Hematological parameters

Blood glucose levels were determined using the method of Nelson and Somogyi (44). Blood samples were deproteinized using zinc sulfate and barium hydroxide, followed by filtration. The absorbance of the resulting supernatant was measured at 540 nm against a blank, and glucose concentration was expressed as mg dL^-^¹.

### Respiratory burst activity

Respiratory burst activity was assessed according to the method of Secombes, as modified by Stasiack and Baumann (45). Briefly, 50 µl of blood was dispensed into wells of U-bottom microtiter plates and incubated at 37 °C for 1 hour to allow cell adhesion. The supernatant was removed, and wells were washed three times with phosphate-buffered saline (PBS). Subsequently, 50 µl of nitroblue tetrazolium (NBT) solution was added and incubated for 1 hour. Cells were fixed with 100% methanol for 2–3 minutes, followed by washing with 30% methanol. After air-drying, 60 µl potassium hydroxide and 70 µl dimethyl sulfoxide were added to solubilize the formazan precipitate. The optical density of the resulting solution was measured at 620 nm using an ELISA plate reader.

### Cortisol

Serum cortisol levels were quantified in all treatment groups using an enzyme-linked immunosorbent assay (ELISA). The analysis was performed with a commercially available Cortisol EIA kit (Cayman Chemical, USA; Catalog No. 500360), following the manufacturer’s instructions. Absorbance was measured using a microplate reader, and cortisol concentrations were calculated accordingly.

### Karyotyping

#### Preparation of reagents, [Colchicine (C_22_H_25_NO_6_)]

A stock solution of colchicine (C_22_H_25_NO_6_; HiMedia) was prepared by dissolving 5 mg in 10 ml of triple-distilled water and stored at 4 °C. A working solution (0.05 mg ml^-^¹) was prepared by diluting 0.5 ml of the stock solution to a final volume of 5 ml with triple-distilled water. The working solution was administered intramuscularly to fish at a dose of 0.05 mg per 100 g body weight, 4 hours prior to sacrifice for tissue collection.

#### Hypotonic saline solution (0.075M KCl)

0.075 M potassium chloride (KCl) solution was prepared by dissolving 0.56 g KCl in 100 ml of triple-distilled water. The solution was freshly prepared prior to use.

#### Tissue collection

Fish were gently removed from the water, blotted dry using clean cotton cloth, and the abdominal surface was disinfected with 70% ethanol. Kidney tissues were excised aseptically and immediately transferred to Petri dishes containing freshly prepared hypotonic KCl solution. The tissues were finely minced immediately after dissection.

### Preparation of metaphase spread

#### Hypotonic treatment and fixation

Minced tissues were incubated in hypotonic KCl solution for 30 minutes at room temperature. The cell suspension was then transferred to centrifuge tubes and centrifuged at 1000 rpm for 10 minutes. The supernatant was discarded, and approximately 5 ml of freshly prepared fixative (methanol:glacial acetic acid, 3:1 v/v) was added. The suspension was mixed thoroughly and centrifuged again under the same conditions. This fixation step was repeated 2–3 times until a clear supernatant and a compact white cell pellet were obtained. The final pellet was resuspended in an appropriate volume of fixative to obtain a uniform cell suspension. A few drops of the suspension were then dropped onto clean, chilled glass slides and air-dried for approximately 1 hour (32).

#### Staining of chromosome

Slides were stained with 4% Giemsa prepared in Sorenson’s phosphate buffer for 30 minutes using a Coplin jar. After staining, slides were rinsed with distilled water, air-dried, and mounted using DPX mounting medium.

#### Histopathology

Gill and liver tissues from control and endosulfan-exposed fish were fixed in neutral buffered formalin for 48 hours. Fixed tissues were washed under running tap water and dehydrated through ascending grades of ethanol. Samples were then cleared in xylene (two changes, 30 minutes each) and embedded in paraffin wax. Sections of 5 µm thickness were obtained using a rotary microtome and mounted on glass slides. Sections were deparaffinized in xylene, rehydrated through graded alcohol series, and rinsed in distilled water. Hematoxylin staining was carried out for 12 minutes, followed by differentiation in 1% acid alcohol and rinsing in tap water. Sections were then dehydrated through graded alcohol (50%, 70%, and 90%) and counterstained with eosin for 4 minutes. After final dehydration in absolute alcohol, sections were cleared in xylene and mounted using DPX. Prepared slides were examined under a light microscope (Olympus CX-31, Japan), and representative photomicrographs were captured for analysis (46).

### Statistics

Data analysis was carried out using SPSS software (version 16.0; SPSS Inc., Chicago, IL, USA). Experimental results are reported as mean ± SE. The distribution of the data and equality of variances were evaluated using the Shapiro-Wilk and Levene’s tests, respectively. Upon confirmation of normality and homoscedasticity, one-way ANOVA was applied to determine treatment effects, and Duncan’s multiple range test (DMRT) was used for *post hoc* comparisons among means. Statistical significance was established at a probability level of p< 0.05.

## Results and discussion

### Acute toxicity and LC_50_

The acute toxicity of endosulfan in freshwater-acclimated juvenile milkfish (*Chanos chanos*) (0 ppt salinity) was evaluated following exposure to a range of concentrations. Cumulative mortality responses at 24, 48, 72, and 96 hours, along with the corresponding LC_50_ values and 95% confidence limits, are presented in **Figure 1** and **Table 1**, respectively. The LC_50_ values for endosulfan at 24, 48, 72, and 96 hours were determined to be 30.88, 29.16, 28.38, and 27.47 µg L^-^¹, respectively. The test compound used in the present study was 99% pure endosulfan, comprising α and β isomers in a ratio of 70:30.

**Figure 1:**
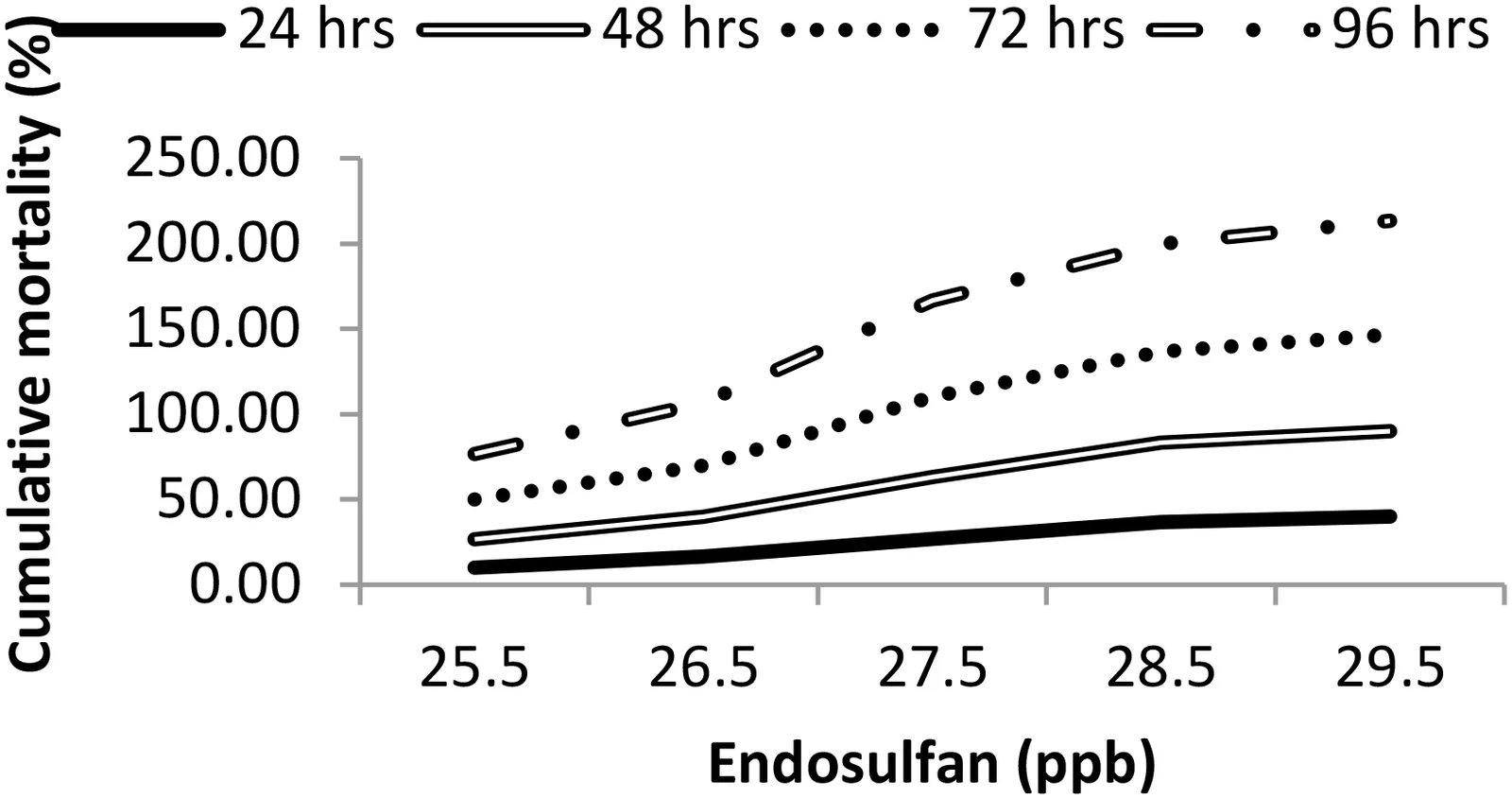
Cumulative mortality (%) of Milk fish (*Chanos chanos*) acclimated to fresh water condition to different concentrations of endosulfan (99% EC) for a period of 96 h (n = 30 for each concentration).

**Table 1:** Median lethal concentration (LC_50_) of Milk fish (*Chanos chanos*) acclimated to fresh water and exposed to different concentrations of endosulfan (99% EC) for a period of 96 h.

Period of exposure (hrs)	R^2^ Value	LC_50_	95 % confidence interval		S-value	Safe level	Intercept	Slope
			Lower level	Upper level				
24	0.750	30.88	29.32	39.67	1.06	7.80	-19.4	0.80
48	0.720	29.16	28.22	32.00			-21.28	0.90
72	0.700	28.38	27.50	30.39			-20.55	0.91
96	0.740	27.47	26.64	28.35			-24.33	1.06

The LC_50_ value is a widely accepted endpoint in acute toxicity studies, representing the concentration of a toxicant that results in 50% mortality of test organisms within a specified exposure period (96 hours). Mortality is commonly employed as a primary indicator in toxicity assessments due to its ease of determination and clear ecological relevance. Comparative LC_50_ values for endosulfan reported in freshwater and brackishwater fish species are summarized in **Table 2** (20, 33, 47–49). To the best of our knowledge, this study provides the first report on the 96-hour LC_50_ of endosulfan in freshwater-acclimated juvenile *C. chanos*. In contrast, our earlier study reported a 96-hour LC_50_ value of 21.09 µg L^-^¹ for juveniles *C. chanos* acclimated to 35 ppt salinity (16). Additionally, Magesh and Kumaraguru (20) reported a substantially lower 96-hour LC_50_ value of 0.566 µg L^-^¹ in milkfish fry (mean body weight: 0.342 g), indicating higher sensitivity in early life stages.

**Table 2:** Acute study reported in fresh and brackish water fish for 96 h LC_50_ of endosulfan.

Test species	Common name	LC50 (µg/L)	End time (hr/system)	Purity	Reference
*Mugil Cephalus*	Flathead mullet	0.09	96	....	Schimmel et al. (1977) (10)
*Labeo rohita*	Rohu (4 g)	2.0 ppb	96	99% EC	Mutthappa et al. (2011)
*Chanos Chanos*	Milk fish (0.342 g)	0.56	96	Technical (35EC %)	Magesh & Kumaraguru (2006) (20)
*Oreochromis mossambicus*	Tilapia (46 g)	3.60	96	99% EC	Kumar et al. (2011) (33)
*Menidia beryllina*	Inland silverside	1.50	96	....	Hemmer et al. (1992) (49)
*Atherinops affinis*	Topsmelt Silverside	1.30	96	....	Hemmer et al. (1992) (49)
*Chanos Chanos*	Milk fish (110 g)	21.09	96	99% EC	Kumar et al., 2016 (16)
*Chanos Chanos*	Milk fish (110 g)	27.47	96	99% EC	This study

### Stress biomarker enzymes

#### Antioxidative enzymes

The activities of key antioxidant enzymes such as catalase (CAT), superoxide dismutase (SOD), and glutathione S-transferase (GST) in the liver, gill, and brain of juvenile *Chanos chanos* exposed to endosulfan are presented in **Table 3** and **4**. A significant difference (p<0.01) was observed between control and treated groups. Exposure to endosulfan resulted in a marked, dose and time dependent elevation in the activities of all three enzymes across the examined tissues. Catalase activity exhibited a pronounced and significant (p<0.01) increase in all tissues. In the liver and gill, CAT activity increased by 198-721% and 153-544%, respectively, in fish exposed to 25.5-29.5 µg L^-^¹ endosulfan compared to controls. Notably, the gill tissue showed the highest sensitivity, with CAT activity increasing by 203-999% under the same exposure conditions. Similarly, SOD activity in the liver, gill, and brain showed a significant (p<0.01) elevation relative to controls, with increases ranging from 90-354%, 88-207%, and 67-161%, respectively, across the tested concentration range. Glutathione S-transferase activity also increased significantly (p<0.01) in all tissues. At the lowest exposure concentration (25.5 µg L^-^¹), GST activity increased by approximately 83%, 76%, and 68% in the liver, gill, and brain, respectively, whereas at the highest concentration (29.5 µg L^-^¹), the increases reached 396%, 229%, and 241%, respectively.

**Table 3:** Catalase and SOD activity in liver, gill and brain tissues of Milk fish (*Chanos chanos*) acclimated to fresh water and exposed to different concentration of endosulfan (99% EC) for a period of 96 h.

Endosulfan (µg/L)	Catalase			SOD		
	Liver	Gill	Brain	Liver	Gill	Brain
0 (Control)	0.73^a^±0.09	0.56^a^±0.09	1.14^a^±0.09	7.81^a^±0.21	9.77^a^±0.87	16.21^a^±2.34
25.5	2.18^b^±0.38	1.69^b^±0.15	2.90^b^±0.15	14.90^b^±2.26	18.41^b^±0.59	27.21^b^±1.44
26.5	2.86^bc^±0.90	2.55^b^±0.68	3.68^b^±0.35	24.32^c^±1.09	20.70^bc^±0.30	36.01^c^±1.94
27.5	3.64^bc^±0.41	4.10^c^±0.45	4.01^c^±0.60	28.30^cd^±1.35	24.80^c^±3.45	37.48^c^±1.40
28.5	4.53^c^±0.67	4.88^c^±0.79	5.72^d^±0.40	30.35^d^±0.46	23.10^c^±1.85	38.91^c^±2.34
29.5	6.01^d^±0.58	6.13^d^±0.83	7.35^e^±0.85	35.47^e^±2.36	30.04^d^±2.40	42.30^d^±3.38
P-Value	p<0.01	p<0.01	p<0.01	p<0.01	p<0.01	p<0.01

Values in the same column with different superscript differ significantly (P < 0.01).

Data expressed as Mean ± SE (N = 6).

Catalase: Units/mg protein, SOD: Super oxide dismutase: Units/mg protein,

**Table 4:** Glutathione-S-transferase activity in liver, gill and brain tissues and AChE activity in brain of Milk fish (*Chanos chanos*) acclimated to fresh water and exposed to different concentration of endosulfan (99% EC) for a period of 96 h.

Endosulfan (µg/L)	GST			AChE
	Liver	Gill	Brain	Brain
0 (Control)	0.12^a^±0.01	0.25^a^±0.07	0.22^a^±0.05	0.46^c^±0.01
25.5	0.23^b^±0.01	0.45^b^±0.02	0.37^b^±0.01	0.33^b^±0.01
26.5	0.28^b^±0.02	0.54^b^±0.01	0.46^bc^±0.02	0.24^a^±0.07
27.5	0.38^c^±0.05	0.60^c^±0.03	0.48^bc^±0.03	0.23^a^±0.01
28.5	0.52^d^±0.06	0.63^c^±0.02	0.59^c^±0.05	0.21^a^±0.02
29.5	0.61^e^±0.03	0.84^d^±0.07	0.75^d^±0.07	0.16^a^±0.02
P-Value	p<0.01	p<0.01	p<0.01	p<0.01

Values in the same column with different superscript differ significantly (P < 0.01).

Data expressed as Mean ± SE (N = 6).

GST: Units/mg protein, AChE: nmole/min/mg protein

Among the evaluated antioxidant enzymes, catalase appeared to be the most responsive and protective against oxidative stress in *C. chanos* following 96-hour exposure to endosulfan. Endosulfan is known to induce oxidative stress through the generation of oxygen free radicals (OFRs), leading to cellular and molecular damage (50, 51). Due to their high reactivity, OFRs can disrupt essential cellular structures and functions, including lipids, proteins, and nucleic acids (52). In response, both enzymatic and non-enzymatic antioxidant defense systems are activated to mitigate oxidative damage (53). The observed elevation in CAT and SOD activities in the liver, gill, and brain suggests an adaptive protective response to endosulfan-induced oxidative stress. The liver, being the primary organ involved in detoxification, plays a crucial role in the biotransformation of endosulfan into metabolites such as endosulfan ether (53, 54). Consequently, alterations in hepatic antioxidant enzyme activities serve as sensitive biomarkers of endosulfan toxicity and oxidative stress in exposed fish.

#### Enzyme of neurotransmitter

A progressive decline in acetylcholinesterase (AChE) activity was observed in the brain of *Chanos chanos* with increasing concentrations of endosulfan. Compared to the control group, AChE activity was significantly inhibited (p<0.01) across all exposure levels (25.5-29.5 µg L^-^¹), with a clear dose-dependent trend (**Table 4**). The extent of inhibition reached 27%, 47%, 48%, 53%, and 65% at 25.5, 26.5, 27.5, 28.5, and 29.5 µg L^-^¹, respectively.

Although AChE inhibition is a well-established biomarker of organophosphate toxicity (55), its mechanistic basis in organochlorine-induced toxicity, particularly endosulfan exposure, warrants further investigation. The degree of AChE inhibition is often correlated with mortality and is widely employed as a sensitive diagnostic indicator of pesticide exposure in aquatic organisms (56, 57). Similar reductions in brain AChE activity following endosulfan exposure have been reported in *Oreochromis mossambicus* (33), supporting the present findings.

#### Glycolytic enzymes

Activities of lactate dehydrogenase (LDH) and malate dehydrogenase (MDH) showed a significant (p< 0.01) and dose-dependent increase in the liver, gill, and brain tissues of fish exposed to endosulfan (**Table 5**). Compared to the control group, LDH activity increased by 131-416%, 81-280%, and 136–327% in the liver, gill, and brain, respectively, across the concentration range of 25.5-29.5 µg L^-^¹. Similarly, MDH activity exhibited a significant (p<0.01) elevation, increasing by 63-242% in the liver, 144-468% in the gill, and 82-314% in the brain under the same exposure conditions.

**Table 5:** LDH and MDH activity in liver, gill and brain tissues of Milk fish (*Chanos chanos*) acclimated to fresh water and exposed to different concentration of endosulfan (99% EC) for a period of 96 h.

Endosulfan (µg/L)	LDH			MDH		
	Liver	Gill	Brain	Liver	Gill	Brain
0 (Control)	0.17^a^±0.02	0.34^a^±0.03	0.18^a^±0.03	0.33^a^±0.08	0.23^a^±0.01	0.22^a^±0.04
25.5	0.39^b^±0.05	0.61^b^±0.06	0.42^b^±0.01	0.54^b^±0.08	0.57^b^±0.04	0.40^b^±0.04
26.5	0.47^bc^±0.04	0.92^c^±0.02	0.54^bc^±0.02	0.67^bc^±0.06	0.87^c^±0.07	0.58^c^±0.02
27.5	0.55^c^±0.06	0.86^c^±0.08	0.53^bc^±0.02	0.82^c^±0.06	0.86^c^±0.11	0.63^cd^±0.06
28.5	0.72^d^±0.04	1.08^d^±0.16	0.72^c^±0.06	0.88^c^±0.07	1.06^cd^±0.21	0.77^d^±0.05
29.5	0.86^e^±0.07	1.28^d^±0.11	0.75^c^±0.18	1.14^d^±0.03	1.33^d^±0.09	0.90^e^±0.09
P-Value	p<0.01	p<0.01	p<0.01	p<0.01	p<0.01	p<0.01

Values in the same column with different superscript differ significantly (P < 0.01).

Data expressed as Mean ± SE (N = 6).

LDH and MDH: units/min/mg protein at 37 ^0^C

The marked increase in LDH activity suggests a shift toward anaerobic metabolism, likely due to impaired oxygen availability at the cellular level, resulting in enhanced lactate production. Elevated MDH activity, a key enzyme of the tricarboxylic acid (TCA) cycle, indicates increased metabolic demand and enhanced flux through energy-producing pathways. The increased production of oxaloacetate may support transamination reactions mediated by AST, thereby facilitating ATP generation to meet heightened physiological stress. The observed elevation in LDH and MDH activities in *C. chanos* corroborates earlier findings. For instance, increased LDH activity following acute endosulfan exposure has been reported in *Oreochromis mossambicus* (33), while enhanced MDH activity has been documented in *Channa punctatus* under sublethal endosulfan exposure (58). These findings collectively suggest that endosulfan induces significant metabolic reprogramming in fish as an adaptive response to toxic stress.

#### Enzymes of protein metabolism and pentose phosphate pathway

Alanine aminotransferase (ALT) and aspartate aminotransferase (AST) activities in the liver and gill increased significantly (p < 0.01) in *Chanos chanos* exposed to endosulfan compared to the control group (**Table 6**). ALT activity in the liver and gill exhibited a marked elevation, ranging from 83–557% and 76–389%, respectively, across the concentration range of 25.5–29.5 µg L^-^¹. Similarly, AST activity increased significantly, with corresponding elevations of 103–343% in the liver and 84–294% in the gill.

**Table 6:** ALT, AST and G6PDH activity in liver and gill tissues of Milk fish (*Chanos chanos*) acclimated to fresh water and exposed to different concentration of endosulfan (99% EC) for a period of 96 h.

Endosulfan (µg/L)	ALT		AST		G6PDH	
	Liver	Gill	Liver	Gill	Liver	Gill
0 (Control)	4.96^a^±0.43	10.94^a^±0.76	6.69^a^±1.37	14.35^a^±0.27	0.09^a^±0.01	0.12^a^±0.01
25.5	9.12^b^±0.48	19.30^b^±1.30	13.63^b^±2.06	26.54^b^±1.90	0.17^b^±0.01	0.31^b^±0.03
26.5	17.74^c^±0.61	31.93^c^±4.10	21.06^c^±1.60	39.85^c^±3.85	0.20^b^±0.01	0.37^b^±0.06
27.5	21.46^cd^±0.45	31.61^c^±0.64	21.23^c^±3.39	34.30^cb^±4.05	0.32^c^±0.03	0.40^b^±0.06
28.5	23.30^d^±2.88	41.42^d^±4.25	26.25^cd^±0.62	46.07^d^±4.95	0.38^d^±0.02	0.47^b^±0.10
29.5	32.66^e^±1.24	53.49^e^±0.56	29.65^d^±1.64	56.58^e^±2.57	0.36^d^±0.01	0.46^b^±0.03
P-Value	p<0.01	p<0.01	p<0.01	p<0.01	p<0.01	p<0.01

Values in the same column with different superscript differ significantly (P < 0.01).

Data expressed as Mean ± SE (N = 6).

ALT: nmole of sodium pyruvate formed/mg protein/min at 37 ^0^C, AST: nmole Oxalo acetate released/min/mg protein at 37 ^0^C, G6PDH: Change in 0.01 OD/min/mg protein

The observed elevation in transaminase activities may indicate enhanced protein catabolism and metabolic reprogramming in response to toxic stress. Increased activities of alanine aminotransferase (ALT) and aspartate aminotransferase (AST) may be associated with enhanced gluconeogenesis to fulfil the increased energy requirements under stress, along with a possible suppression of *de novo* protein synthesis. In addition, elevated transaminase activities can reflect disruption of hepatocellular membrane integrity, resulting in the release of intracellular enzymes into the surrounding tissues and circulation. The keto acids generated during transamination may subsequently enter the tricarboxylic acid (TCA) cycle and contribute to ATP generation to meet the elevated energy demands during toxic exposure. In the present study, the significant increases in ALT and AST activities in the liver and gill tissues, accompanied by pronounced histopathological alterations, suggest substantial physiological stress and tissue injury in endosulfan-exposed fish. Similar alterations in transaminase activities have been documented in *Chanos chanos* following acute endosulfan exposure (3, 16) and in *Labeo rohita* subjected to chronic exposure (31). Comparable responses have also been reported in *Anguilla anguilla* exposed to deltamethrin (59), further supporting the potential utility of ALT and AST as biochemical indicators of pesticide-induced physiological stress and tissue damage.

#### Enzymes of pentose phosphate pathway

Glucose-6-phosphate dehydrogenase (G6PDH) activity showed a significant (p < 0.01) increase in both liver and gill tissues compared to the control group (**Table 6**). In the liver, G6PDH activity increased from 87% to 292%, while in the gill it ranged from 147% to 267% across the tested concentrations (25.5–29.5 µg L^-^¹). A progressive increase in enzyme activity was observed with rising endosulfan concentrations, indicating a dose-dependent response.

G6PDH catalyzes the rate-limiting step of the pentose phosphate pathway and serves as a primary source of NADPH, which is essential for biosynthetic processes and maintenance of cellular redox homeostasis (60). NADPH plays a critical role in sustaining reduced glutathione (GSH) levels, thereby supporting antioxidant defense mechanisms against oxidative stress (61). The elevated G6PDH activity observed in this study suggests an increased demand for NADPH during endosulfan-induced stress, likely to counteract enhanced oxidative burden and to maintain cellular homeostasis (62, 63). These findings are consistent with our previous observations in *Oreochromis mossambicus* following acute endosulfan exposure (3), further reinforcing the involvement of the pentose phosphate pathway in adaptive stress responses.

#### Cortisol, blood glucose and NBT (respiratory burst activity)

Serum cortisol, blood glucose levels, and respiratory burst activity of phagocytes assessed via nitroblue tetrazolium (NBT) reduction are presented in **Table 7**. A significant (p<0.01) increase in serum cortisol was observed in endosulfan-exposed fish, with elevations of 13% and 43% at 25.5 and 29.5 µg L^-^¹, respectively. In contrast, a significant (p < 0.01), dose and time-dependent suppression of immune function was evident, as indicated by reduced NBT activity, alongside a marked increase in blood glucose levels. Phagocytic activity declined significantly (p<0.01) in fish exposed to increasing concentrations of endosulfan, with reductions of 15%, 35%, 52%, 55%, and 60% at 25.5, 26.5, 27.5, 28.5, and 29.5 µg L^-^¹, respectively. Concurrently, blood glucose levels increased by 41–78% relative to control fish, indicating a pronounced stress response.

**Table 7:** Level of blood glucose, cortisol and NBT activity of Milk fish (*Chanos chanos*) acclimated to fresh water and exposed to different concentration of endosulfan (99% EC) for a period of 96 h.

Endosulfan (µg/L)	Blood Glucose	Cortisol	NBT
0 (Control)	25.46^a^±0.56	105.47^a^±4.39	1.00^d^±0.02
25.5	36.01^b^±2.87	119.62^b^±1.05	0.84^c^±0.05
26.5	41.45^bc^±1.17	126.80^c^±0.65	0.64^b^±0.06
27.5	40.36^bc^±2.56	134.83^d^±0.58	0.48^a^±0.02
28.5	43.00^c^±1.02	141.83^e^±1.16	0.45^a^±0.02
29.5	45.48^c^±0.62	151.36^f^±1.48	0.39^a^±0.01
P-Value	p<0.01	p<0.01	p<0.01

Values in the same column with different superscript differ significantly (P < 0.01).

Data expressed as Mean ± SE (N = 6).

Blood Glucose: mg/dl, Cortisol: ng/ml, NBT: OD at 630 nm

Cortisol is the principal corticosteroid in teleost fish and plays a central role in stress physiology, particularly in osmoregulation and energy metabolism (64). It is a key indicator of activation of the hypothalamo-pituitary-interrenal (HPI) axis, the primary neuroendocrine pathway mediating stress responses in fish (65). Elevated cortisol levels facilitate the mobilization of energy reserves, including glucose, lipids, and fatty acids, thereby enabling the organism to cope with increased metabolic demands under stress. The significant elevation of cortisol observed in this study reflects an adaptive physiological response to endosulfan exposure. Similar increases in cortisol levels have been reported in *Oreochromis mossambicus* and *Labeo rohita* under endosulfan-induced stress (33, 34).

The NBT assay is widely used to evaluate respiratory burst activity, which reflects the ability of phagocytes to generate reactive oxygen species (ROS), such as superoxide anions, hydrogen peroxide, and hydroxyl radicals, during pathogen defense (66). These ROS play a crucial role in microbial killing and immune competence (67). Therefore, enhanced NBT activity is typically associated with improved immune function (68). However, the significant reduction in NBT activity observed in the present study suggests immunosuppression in *C. chanos* under endosulfan exposure. A similar decline in NBT activity has been reported in *Oreochromis mossambicus* following acute endosulfan exposure (33). Notably, limited information is available regarding the combined effects of endosulfan on cortisol elevation and immune suppression in milkfish, highlighting the relevance of the present findings.

Blood glucose is a key metabolic indicator and is commonly used as a biomarker of stress in fish exposed to environmental stressors, including pollutants (69). The observed increase in glucose levels (41–78%) in response to endosulfan exposure indicates enhanced metabolic demand and stress-induced hyperglycemia. This response is likely mediated by increased glycogenolysis and gluconeogenesis, involving the conversion of non-carbohydrate substrates such as amino acids into glucose (70). Additionally, stress-induced secretion of catecholamines may further stimulate glycogen breakdown, contributing to elevated blood glucose levels (71). Collectively, the observed alterations in cortisol, immune function, and glucose metabolism indicate that endosulfan exposure induces a pronounced stress response and compromises immunological competence in *C. chanos*.

##### Top of form

##### Bottom of form

### Karyotyping

Acute exposure (96 h) to endosulfan induced marked chromosomal aberrations in the kidney tissues of *Chanos chanos*, as illustrated in **Figures 2A–D**). Control fish exhibited normal chromosomal morphology, whereas exposed groups showed a range of structural abnormalities. At 27.5 µg L^-^¹, chromosomes displayed terminal stickiness (**Figure 2B**), while clumping was evident at 28.5 µg L^-^¹ (**Figure 2C**). At the highest concentration (29.5 µg L^-^¹), severe aberrations were observed, including chromosomal end-joining, fragmentation, and attenuation (**Figure 2D**).

**Figure 2:**
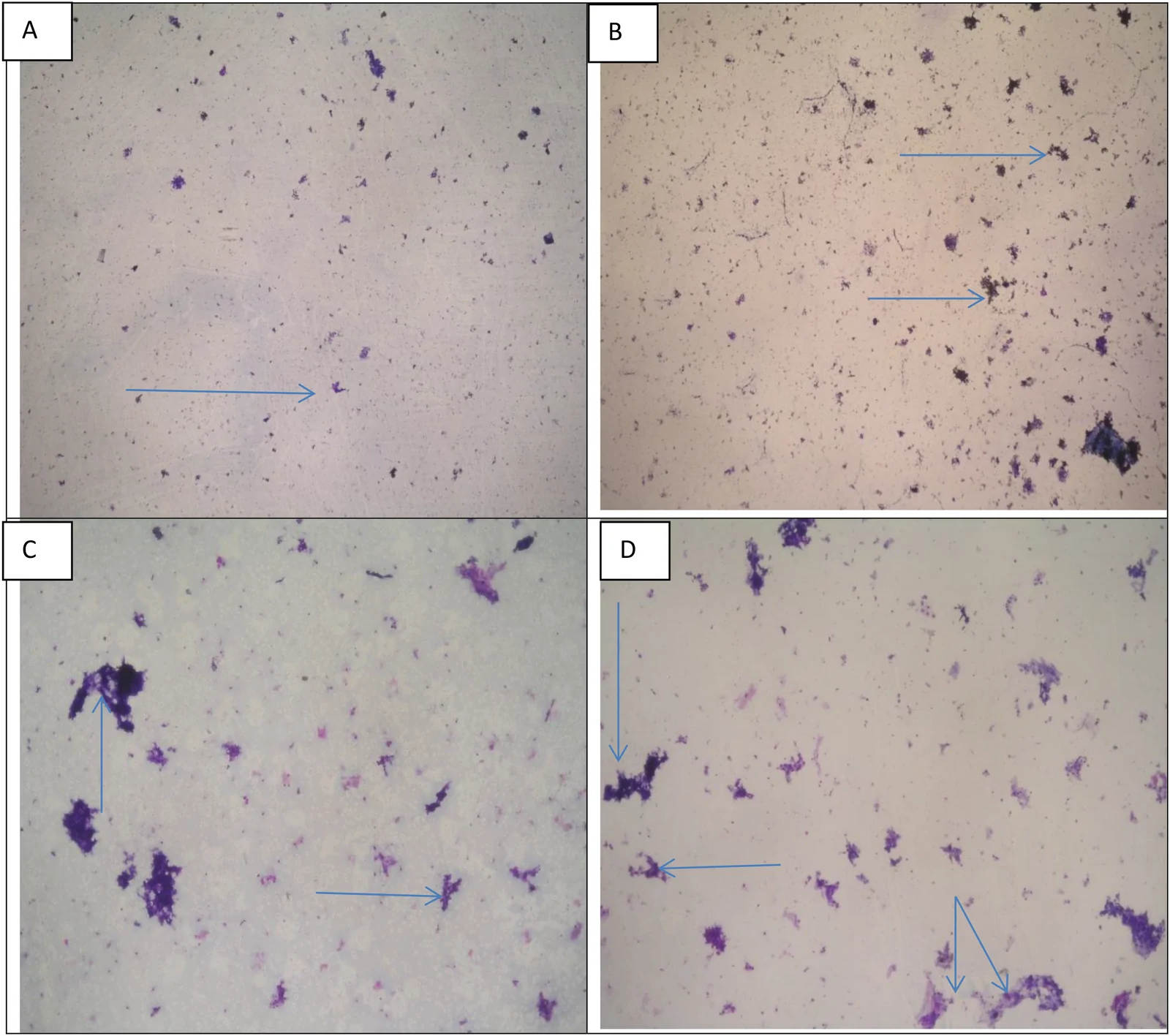
Karyotyping of kidney tissue in milk fish (Chanos chanos). **(A)** Unexposed group Normal chromosomes are shown **(B)**. Endosulfan-exposed group: aberrated chromosomes are observed in 27.5µg/L of endosulfan exposed were showed stickiness among the chromosome end and **(C)** Clumping among the chromosome at 28.5 µg/L. **(D)** The chromosomes were also found that end to end joining, fragmented and attenuated after 96 h exposure of endosulfan at 29.5µg/L of endosulfan exposure.

These cytogenetic alterations indicate that endosulfan exerts genotoxic effects, directly compromising chromosomal integrity. Such abnormalities likely arise from the clastogenic properties of the pesticide, which may interfere with DNA replication, repair mechanisms, and protein synthesis (32, 72). Similar chromosomal aberrations, including stickiness, clumping, and fragmentation, have been reported in *Cirrhinus mrigala* following butachlor exposure (73), as well as in *Labeo rohita* under prolonged endosulfan exposure (32). The observed disruptions in chromosomal structure suggest that endosulfan may inhibit normal cell division processes, thereby affecting genetic stability.

### Histopathology study

Histopathological changes in the gills of *Chanos chanos* following endosulfan exposure are illustrated in **Figure 3A-F**. Control fish exhibited normal gill architecture, characterized by well-defined primary and secondary lamellae with intact chloride and pillar cells (**Figure 3A**). Exposure to 25.5 µg L^-^¹ endosulfan resulted in epithelial hyperplasia of the primary lamellae and aneurysmal dilation of the secondary lamellae (**Figure 3B**). At 26.5 µg L^-^¹, pronounced aneurysms and thickening of the primary lamellar epithelium were observed (**Figure 3C**). Intermediate concentrations (27.5 and 28.5 µg L^-^¹) induced leukocyte infiltration and further epithelial hyperplasia (**Figures 3D, E**). At the highest concentration (29.5 µg L^-^¹), severe structural damage was evident, including shortening and fusion of secondary lamellae due to excessive epithelial proliferation (**Figure 3F**).

**Figure 3:**
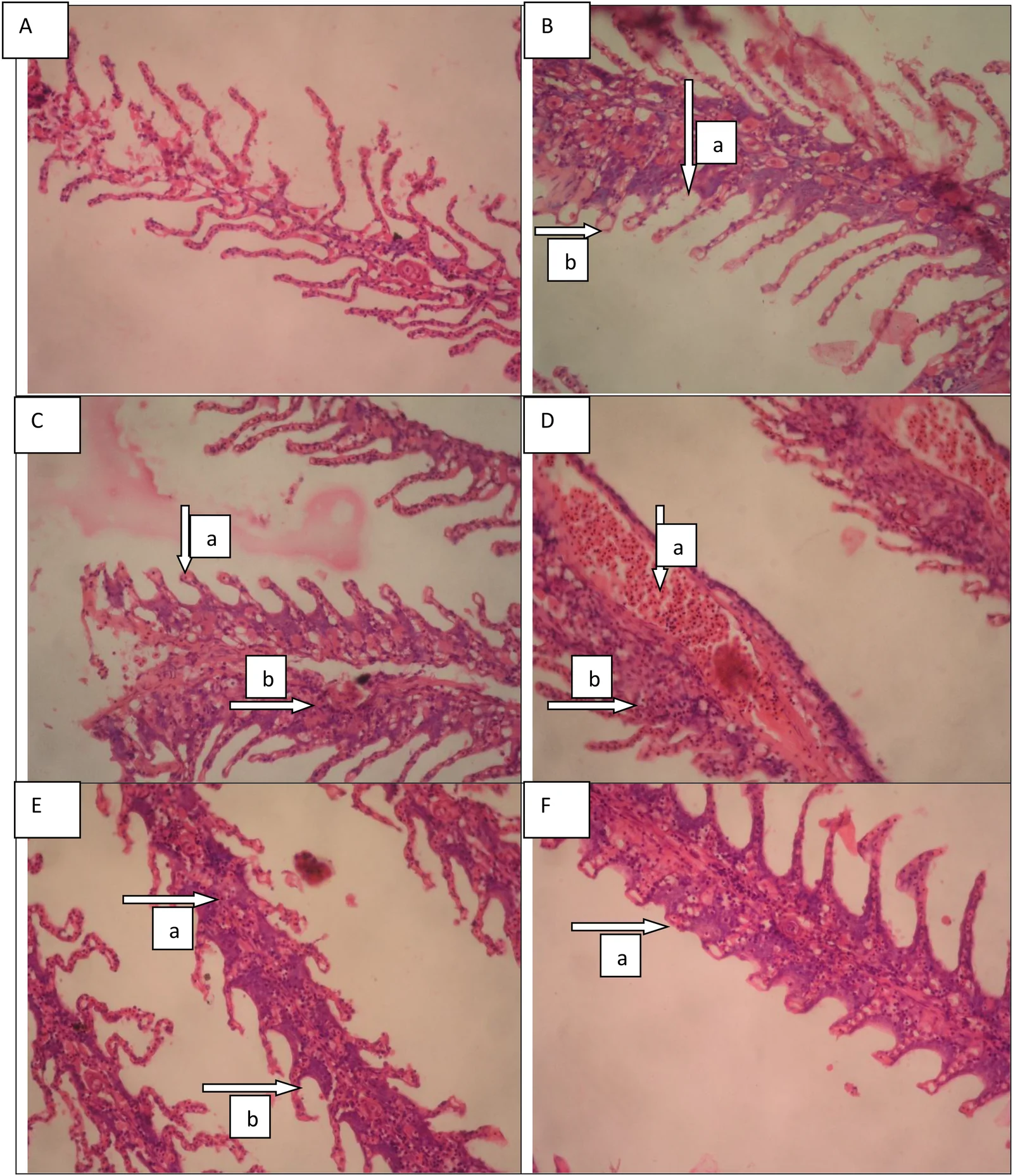
Effect of acute endosulfan exposure on the histological alteration in the gill of Milk fish (*Chanos chanos*). **(A)** Control (0 µg/L) showing normal appearing primary and secondary lamellae with chloride cells and pillar cells. **(B)** 25.5 µg/L endosulfan exposed group exhibiting (a) primary lamellar epithelial hyperplasia (b) thickening of aneurism in secondary lamellae **(C)** 26.5 µg/L endosulfan exposed group showing (a) aneurism in secondary lamellae (b) thickening of primary lamellar epithelial. **(D, E)** 27.5 and 28.5 µg/L endosulfan exposed group (a) infiltration of leucocytes (b) hyperplasia of primary lamellae epithelial cell **(F)** 29.5 µg/L endosulfan exposed group showing (a) shortening of secondary lamellae and fusion of secondary lamellae due to lamellar epithelial (All histology figures were taken in 40X magnification).

These observations indicate that the gill is a primary target organ for endosulfan toxicity. Due to their direct contact with the aquatic environment, gills are highly sensitive indicators of water quality and pollutant exposure (74, 75). As the principal site for respiration, osmoregulation, and excretion, structural damage to gill tissues can significantly impair oxygen uptake, ionic balance, and nitrogenous waste elimination. In the present study, lesions such as epithelial hyperplasia, aneurysmal dilation, lamellar fusion, and tissue degeneration were consistently observed, particularly at higher concentrations (28.5 and 29.5 µg L^-^¹), where severe architectural disruption and atrophy of primary filaments were evident. These findings are consistent with previous reports in *Oreochromis mossambicus* (34), *Oncorhynchus mykiss*, *Cichlasoma dimerus* (76), and catfish species (77) exposed to endosulfan. Such damage is likely to compromise gas exchange efficiency and disrupt osmoregulatory and excretory functions (78, 79).

Histopathological alterations in the liver of *C. chanos* are presented in **Figure 4** (A–E). Control fish displayed normal hepatic architecture, with well-organized hepatocytes and intact nuclei and nucleoli (**Figure 4A**). Fish exposed to moderate concentrations of endosulfan (26.5–28.5 µg L^-^¹) exhibited cellular vacuolation, leukocyte infiltration, and progressive necrosis of hepatocytes (**Figures 4B–D**). At the highest concentration (29.5 µg L^-^¹), severe hepatic damage was observed, characterized by extensive necrosis and loss of cellular integrity (**Figure 4E**).

**Figure 4:**
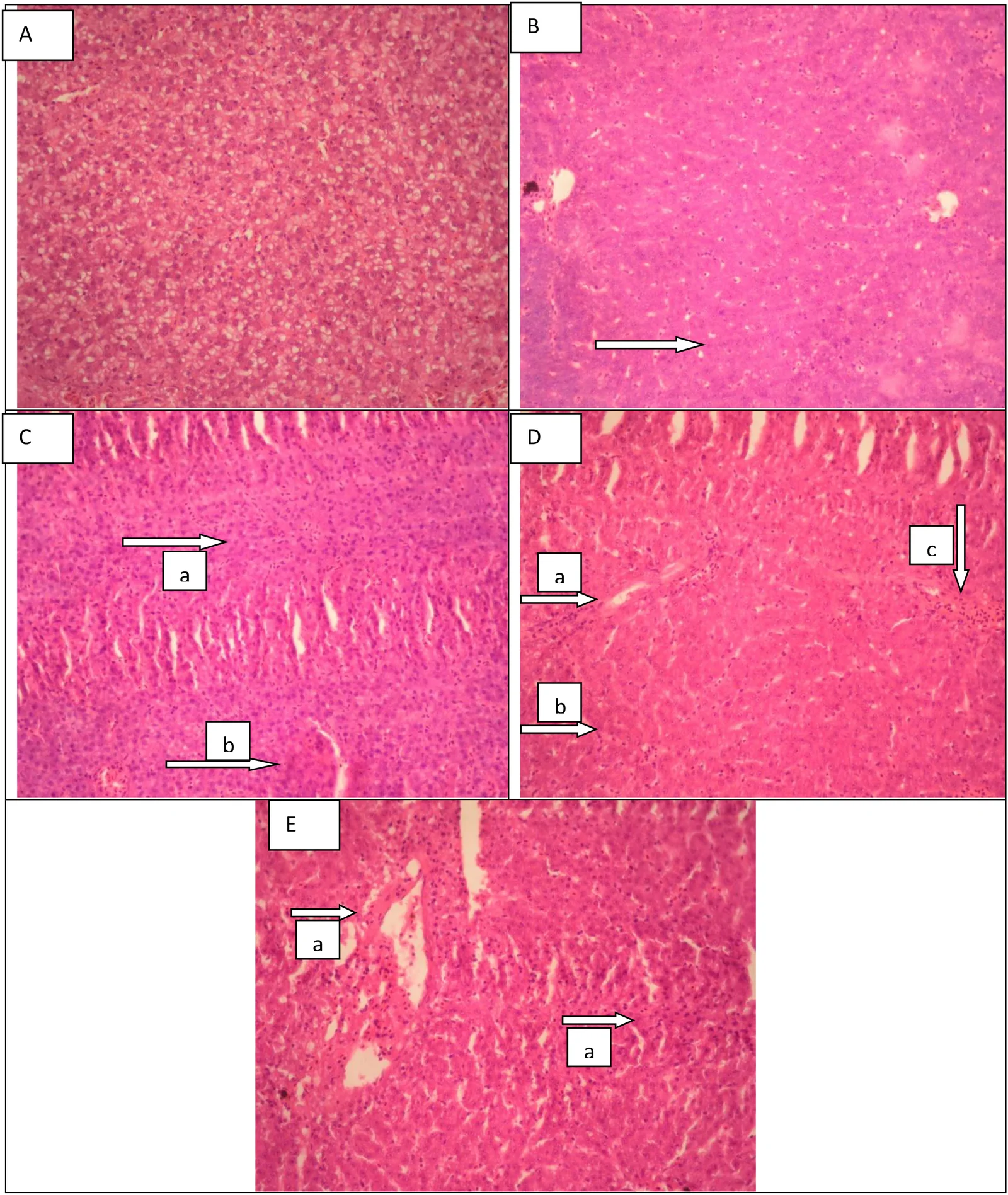
Effect of acute endosulfan exposure on the histological alteration in the liver of Milk Fish (*Chanos chanos*). **(A)** control (0 µg/L) exhibiting normal architecture of hepatic cells with intact nucleus and nucleolus, **(B)** 26.5 µg/L endosulfan exposed group showing vacuolation **(C)** 27.5 µg/L endosulfan exposed group showing (a) infiltration of leucocytes (b) necrosis of liver cells **(D)** 28.5 µg/L endosulfan exposed group exhibiting (a) vacuolation (b) area of congestion (c) infiltration of leucocytes **(E)** 29.5 µg/L endosulfan exposed group showing (a) necrosis of hepatic cells (b) vacuolation. (All histology figures were taken in 40X magnification).

The liver plays a central role in the detoxification of xenobiotics and is therefore highly susceptible to toxic insults (80). Histopathological alterations in hepatic tissue are widely recognized as reliable biomarkers of environmental stress and contaminant exposure. Although the liver possesses significant detoxification capacity, excessive toxicant burden can overwhelm its regulatory mechanisms, leading to structural and functional impairment (81).

In the present study, the progression from mild alterations (vacuolation and leukocyte infiltration) to severe necrosis at higher exposure levels indicates a clear dose-dependent hepatotoxic effect of endosulfan. Similar pathological features, including hepatocellular degeneration and necrosis, have been reported in *Puntius conchonius* (82), *Oncorhynchus mykiss* (83), *Cichlasoma dimerus* (76), and *Oreochromis mossambicus* (34) following pesticide exposure. These structural alterations are likely to impair hepatic function, potentially leading to systemic physiological dysfunction, as suggested in earlier studies (81, 83). Although quantitative frequencies of chromosomal abnormalities and histopathological lesion scores were not determined in the present study, the observed tissue-level alterations were assessed qualitatively based on the histopathological examination of the selected tissues. Representative photomicrographs have been provided to demonstrate the major structural alterations observed in the treatment groups. Therefore, quantitative scoring of lesion severity was beyond the scope of the present investigation and may be considered in future studies to provide a more detailed assessment of tissue damage.

## Conclusion

The present study demonstrates that endosulfan exhibits pronounced acute toxicity to freshwater-acclimated *Chanos chanos*. Exposure to sublethal concentrations resulted in significant alterations in biochemical and metabolic profiles, induction of oxidative stress, inhibition of neurophysiological function, genotoxic effects, and severe histopathological damage in vital organs. These findings highlight the potential ecological risk posed by endosulfan to aquatic organisms, particularly under acute exposure scenarios. The observed multi-level biological disruptions underscore the suitability of the evaluated biomarkers for assessing pesticide-induced stress in fish. Although the study provides critical insights into the toxicological effects of endosulfan, certain limitations and uncertainties remain, particularly with respect to long-term exposure dynamics and interactions with other environmental stressors. Therefore, comprehensive risk assessments considering cumulative and synergistic effects should be undertaken prior to the widespread application of such pesticides in agricultural practices.

## Data Availability

All relevant data is contained within the article. The original contributions presented in the study are included in the article, further inquiries can be directed to the corresponding author.

## References

[1] Boahen E Owusu L Adjei-Anim SO . A comprehensive review of emerging environmental contaminants of global concern. Discover Environ. (2025) 3:144. doi: 10.1007/s44274-025-00259-x30311153

[2] Kumar N Chandan NK Bhushan S Singh DK Kumar S . Health risk assessment and metal contamination in fish, water and soil sediments in the East Kolkata Wetlands, India, Ramsar site. Sci Rep. (2023) 13:1546. doi: 10.1038/s41598-023-28801-y 36707609 PMC9883242

[3] Kumar N Gupta S Chandan NK Aklakur M Pal AK Jadhao SB . Lipotropes protect against pathogen-aggravated stress and mortality in low dose pesticide-exposed fish. PloS One. (2014) 9:e93499. doi: 10.1371/journal.pone.0093499 24690771 PMC3972094

[4] Bashir I Lone FA Bhat RA Mir SA Dar ZA Dar SA . Concerns and threats of contamination on aquatic ecosystems. Bioremediation Biotechnol. (2020) 27:1–26. doi: 10.1007/978-3-030-35691-0_128118817

[5] Ansari I El-Kady MM Mahmoud AED Arora C Verma A Rajarathinam R . Persistent pesticides: Accumulation, health risk assessment, management and remediation: An overview. Desalin Water Treat. (2024) 317:100274. doi: 10.1016/j.dwt.2024.10027442574925

[6] Dalela RC Bhatnagar MC Tyagi SR Verma AK . Histological damage of gill in *Channa gachus* after acute and subacute exposure to endosulfan and Roger. Microscopic (Wien). (1979) 35:301–7. 547199

[7] Ferrando MD Andrew E . Effect of temperature and exposure time and other water parameters on acute toxicity of endosulfsan on European eel *Anguilla Anguilla*. J Environ Sci Health. (1989) 24:219–24.

[8] Gimeno L Ferrando MD Sanchez S Gimeno LO Andreu E . Pesticide effects on eel metabolism. Eco Environ Saf. (1995) 31:153–7. doi: 10.1006/eesa.1995.1056 8521781

[9] Romeo F Quijano MD . Risk assessment in a third world reality: an endosulfan case history. Int J Occup Environ Health. (2000) 6:312–7. doi: 10.1179/oeh.2000.6.4.312 11114124

[10] Schimmel SC Patrickk JJ Wilson AJ . Acute toxicity to and Bio-concentration of endosulfan by estuarine animals. In: Mayer FL Hamelink JL , editors. Aquatic Toxicology and Hazard Evaluation ASTM-STP 634. American Society for testing and materials, Washington DC (1977). p. 241–52.

[11] Kamijima M Casida JE . Regional modification of [3H] Ethynyl bicycle ortho benzoate binding in mouse brain GABAA receptor by endosulfan, fipronil, and avermectin B1a. Toxicol Appl Pharmacol. (2000) 163:188–94. 10.1006/taap.1999.8865 10698677

[12] Stanley KA Curtis LR Simonich SLM Tanguay RL . Endosulfan I and endosulfan sulfate disrupts zebrafish embryonic development. Aquat Toxicol. (2009) 95:355–61. doi: 10.1016/j.aquatox.2009.10.008 19883949 PMC2787831

[13] Jayaraj R Megha P Sreedev P . Organochlorine pesticides, their toxic effects on living organisms and their fate in the environment. Interdiscip Toxicol. (2017) 9:90–100. doi: 10.1515/intox-2016-0012 28652852 PMC5464684

[14] Bhattacharya B Sarkar S Mukherjee N . Organochlorine pesticide residues in sediments of a tropical mangrove estuary, India; implications for monitoring. Environ Internat. (2003) 29:587–92. doi: 10.1016/s0160-4120(03)00016-3 12742401

[15] FAO . Chanos Chanos. In Cultured Aquatic Species Fact Sheets. Text by Nelson, a.L. & Marygrace C.Q. Edited and Compiled by Valerio Crespi and Michael New. CD-ROM (Multilingual). Rome: FAO (2009).

[16] Kumar N Ambasankar K Krishnani KK Gupta SK Bhushan S Minhas PS . Acute toxicity, biochemical and histopathological responses of endosulfan in *Chanos chanos*. Ecotoxicology Environ Saf. (2016) 131:79–88. doi: 10.1016/j.ecoenv.2016.05.013 27213563

[17] FAO . Yearbook Fishery and Aquaculture Statistics: Pp 30. Rome: FAO (2012).

[18] Dildar T Cui W Ikhwanuddin M Ma H . Aquatic organisms in response to salinity stress: Ecological impacts, adaptive mechanisms, and resilience strategies. Biol (Basel). (2025) 14:667. doi: 10.3390/biology14060667 40563918 PMC12189284

[19] Palanikumar L Kumaraguru AK Ramakritinan CM Anand M . Biochemical response of anthracene and benzo pyrene in milkfish *Chanos chanos*. Eco Environ Saf. (2012) 75:187–97. doi: 10.1016/j.ecoenv.2011.08.028 21944957

[20] Magesh S Kumaraguru AK . Acute toxicity of endosulfan to milk fish, *Chanos chanos*, of the southeast coast of India. Bull Environ Contamin Toxicol. (2006) 79:622–8. doi: 10.1007/s00128-006-0965-3 16688544

[21] Kumar N Minhas PS Ambasankar K Krishnani KK . Dietary lecithin potentiates thermal tolerance and cellular stress protection of milk fish (*Chanos chanos*) reared under low dose endosulfan induced stress. J Therm Biol. (2014) 46:40–6. doi: 10.1016/j.jtherbio.2014.10.004 25455939

[22] Morris JJ Rose AL Lu Z . Reactive oxygen species in the world ocean and their impacts on marine ecosystems. Redox Biol. (2022) 52:102285. doi: 10.1016/j.redox.2022.102285 35364435 PMC8972015

[23] Livingstone DR Garcia-Martinez P Michel X Narbonne JF OHara S Ribera D . Oxyradical generation as a pollution-mediated mechanism of toxicity in the common mussel, *Mytilus edulis L* and other molluscs. Funct Ecol. (1990) 4:415–24. doi: 10.2307/2389604

[24] Halliwell B Gutteridge JMC . Free Radicals in Biology and Medicine. Oxford: Oxford University Press (1999). p. 543.

[25] Orbea M Ortiz-Zarragoitia SM Porte C Cajaraville MP . Antioxidant enzymes and peroxisome proliferation in relation to contaminant body bordens of PAHs and PCBs in bivalve molluscs, crabs and fish from the Urdaibai and Plentzia estuaries (Bay of Biscay. Aqua Toxicol. (2002) 58:75–98. doi: 10.1016/s0166-445x(01)00226-0 12062156

[26] Eliska S Veronika P Hana K Martin P Zdenka S . The effect of praziquantel applied per os on selected haematological and biochemical indices in common carp (Cyprinus carpio L.). Fish Physiol Biochem. (2009) 35:599–605. doi: 10.1007/s10695-008-9269-3 19031105

[27] Firat O Hikmet Y Cogun T Yüzereroglu A Gok G Firat O . A comparative study on the effects of a pesticide (cypermethrin) and two metals (copper, lead) to serum biochemistry of Nile tilapia, *Oreochromis niloticus*. Fish Physiol Biochem. (2011) 37:657–66. doi: 10.1007/s10695-011-9466-3 21229307 PMC3146979

[28] Diamantino TC Almeida E Soares AMVM Guilhermino L . Lactate dehydrogenase activity as an effect criterion in toxicity tests with *Daphnia magna* straus. Chemosphere. (2001) 45:556–60. doi: 10.1016/S0045-6535(01)00029-7 11680751

[29] Kumar N Thorat ST Reddy KS . Multi biomarker approach to assess manganese and manganese nanoparticles toxicity in Pangasianodon hypophthalmus. Sci Rep. (2023) 13:8505. doi: 10.1038/s41598-023-35787-0 37231182 PMC10213040

[30] APHA . Standard Methods for the Examination of Water and Wastewater. 20th Editions. Washington, DC: American Public Health Association (1998).

[31] Kumar N Jadhao SB Chandan NK Akhlak M Rana RS . Methyl donors potentiates growth, metabolic status and neurotransmitter enzyme in Labeo rohita fingerlings exposed to endosulfan and temperature. Fish Physiol Biochem. (2012) 38:1343–53. doi: 10.1007/s10695-012-9622-4 22367487

[32] Kumar N Jadhao SB Chandan NK Rana RS . Dietary choline, betaine and lecithin mitigates endosulfan induced stress in *Labeo rohita* fingerlings. Fish Physiol Biochem. (2011) 38:989–1000. doi: 10.1007/s10695-011-9584-y 22160664

[33] Kumar N Prabhu AJ Pal AK Remya S Aklakur M Rana RS . Anti-oxidative and immuno-hematological status of Tilapia (*Oreochromis mossambicus*) during acute toxicity test of endosulfan. Pest Biochem Physiol. (2011) 99:45–52. doi: 10.1016/j.pestbp.2010.10.00342574925

[34] Kumar N Sharma R Tripathi G Kumar K Dalvi RS Krishna G . Cellular metabolic, stress and histological response on exposure to acute toxicity of endosulfan in Tilapia (Oreochromis mossambicus). Environ Toxicol. (2014). doi: 10.1002/tox.22026 25060992

[35] Trevors J . A basic programme for estimating LC50 values using IBM- PC. Bull Environ Contam Toxicol. (1986) 37:18–26. doi: 10.1007/BF01607723 3755069

[36] Misra HP Fridovich I . The role of superoxide anion in the autoxidation of epinephrine and a simple assay for superoxide dismutase. J Biol Chem. (1972) 247:3170–5. doi: 10.1016/s0021-9258(19)45228-9 4623845

[37] Takahara S Hamilton BH Nell JV Kobra TY Ogura Y Nishimura ET . Hypocatalesemia, a new generis carrier state. J Clin Invest. (1960) 29:610–9. doi: 10.1172/JCI104075 PMC293346 13836629

[38] Habing WH Pabst MN Bjacoby W Glutathion S . Transferase, the first enzymatic step in mercatpopunc acid formation. J Biol Chem. (1974) 249:7130–8. 4436300

[39] Hestrin S . The reaction of acetyl choline esters and other carboxylic acid derivatives with hydroxylamine and its analytical application. J Biol Chem. (1949) 180:249–61. doi: 10.1016/s0021-9258(18)56740-5 18133390

[40] Wroblewski L LaDue JS . Lactic dehydrogenase activity in blood. Proc Soc Exp Biol Med. (1955) 90:210–3. doi: 10.3181/00379727-90-21985 13273400

[41] Ochoa S . Malic dehydrogenase and ‘Malic’ enzyme. In: Coloric SP Kaplan N , editors. Methods of Enzymology, Vol I. Academic Press, New York (1955). p. 735–45.

[42] Wootton IDP . Microanalysis in Medical Biochemistry. London: J & A Churchill Ltd (1964) p. 101–3.

[43] De Moss RD . Glucose-6-phosphate and 6-phosphogluconic dehydrogenase from *Leuconostoc mesenteroides*. In: Colowick SP Kalpan NO , editors. Methods in Enzymology, Vol I. Academic Press Inc, New York (1955). p. 328–32.

[44] Nelson N . A photometric adaptation of the Somogyi method for the determination of glucose. J Biol Chem. (1944) 153:375–80. doi: 10.1016/s0021-9258(18)71980-7

[45] Stasiack AS Bauman CP . Neutrophil activity as a potent indicator for concomitant analysis. Fish Shellfish Immunol. (1996) 37:539. doi: 10.1006/fsim.1996.0050

[46] Roberts RJ . Nutritional pathology of teleosts. In: Roberts RJ , editor. Fish Pathology. Bailliere Tindall, London (1989). p. 337–62.

[47] Shimmel SC Patrick JJ Wilson AJ . Acute toxicity to and bioconcentration of endosulfan by estuarine animals aquatic toxicology and hazard evaluation. Am Soc Testing Materials. (1977) 634:241–52. doi: 10.1520/STP32403S

[48] Muthappa NA Jadhao SB Gupta S . Acute toxicity of endosulfan and its effect on activity of some enzymes in Labeo rohita fingerlings. Nat Acad Sci Lett India. (2011) 34:211–8.

[49] Hemmer MJ Middaugh DP Comparetta V . Comparative acute sensitivity of larval topsmelt, Atherinops affinis and inland silverside, Menidia beryllina to 11 chemicals. Environ Toxicol Chem. (1992) 11:401–8. doi: 10.1897/1552-8618(1992)11[401:casolt]2.0.co;2

[50] Shao B Zhu L Dong M Wang J Wang J Xie H . DNA damage and oxidative stress induced by endosulfan exposure in zebrafish (Danio rerio). Ecotoxicology. (2012) 21:1533–40. doi: 10.1007/s10646-012-0907-2 22535316

[51] Kumar N Ambasankar K Dalvi RS Aklakur M Chandan NK Jamwal A . Dietary lecithin ameliorates endosulfan-induced stress responses and promotes growth, immunity, and disease resistance in fingerlings of the milkfish, Chanos chanos. Aquaculture. (2025) 598:741953. doi: 10.1016/j.aquaculture.2024.74195342574925

[52] Chandimali N Bak SG Park EH Lim H-J Won Y-S Kim E-K . Free radicals and their impact on health and antioxidant defenses: a review. Cell Death Discov. (2025) 11:19. doi: 10.1038/s41420-024-02278-8 39856066 PMC11760946

[53] Irato P Santovito G . Enzymatic and non-enzymatic molecules with antioxidant function. Antioxidants (Basel). (2021) 10:579. doi: 10.3390/antiox10040579 33918542 PMC8070535

[54] Murali M Shivanandappa T . Endosulfan causes oxidative stress in the liver and brain that involves inhibition of NADH dehydrogenase and altered antioxidant enzyme status in rat. Ecotoxicol Environ Saf. (2022) 239:113593. doi: 10.1016/j.ecoenv.2022.113593 35567928

[55] Sanchez-Hernandez JC Walker CH . *In vitro* and *in vivo* cholinesterase inhibition in Lacertides by phosphonate- and phosphorothioate- type organophosphates. Pest Biochem Physiol. (2000) 67:1–12. doi: 10.1006/pest.1999.247121632986

[56] Shinde A Sharma R Kumar P Kumar T Reddy KS Kumar N . Combined effect of mercury and ammonia toxicity and its mitigation using selenium nanoparticles in fish. Aquat Toxicol. (2025) 280:107270. doi: 10.1016/j.aquatox.2025.107270 39954588

[57] Rokade A Thorat ST Chandramore K Reddy KS Kumar N . Integrating immunity, anti-oxidative status and gene regulation against nickel and high temperature stress in fish: Selenium nanoparticles for mitigation. Environ Sci pollut Res. (2025) 32:3987–4003. doi: 10.1007/s11356-025-35947-x 39843820

[58] Sarma K Pal AK Sahu NP Ayyappan S Baruah K . Dietary high protein and vitamin c mitigates endosulfan toxicity in the spotted murrel, Channa punctatus (Bloch, 1793). Sci Total Environ. (2009) 407:3668–73. doi: 10.1016/j.scitotenv.2009.02.031 19324396

[59] Balint T Ferencsy J Katai J Kiss I Kraczer L Kufcsak O . Similarities and differences between the massive eel (*Anguilla Anguilla* L.) devastations that occurred in Lake Balaton in 1991 and 1995. Eco Environ Saf. (1997) 37:17–23. doi: 10.1006/eesa.1996.1509 9212331

[60] Kletzien RF Harris PKW Foellmi LA . Glucose-6- phosphate dehydrogenase: a “housekeeping” enzyme subject to tissue-specific regulation by hormones, nutrients, and oxidant stress. FASEB J. (1994) 8:174–81. 10.1096/fasebj.8.2.8119488 8119488

[61] Leopold JA Loscalzo J . Cyclic strain modulates resistance to oxidant stress by increasing G6PDH expression in smooth muscle cells. Am J Physiol. (2000) 279:H2477–85. doi: 10.1152/ajpheart.2000.279.5.h2477 11045985

[62] Mondal A Mukherjee S Upadhyay P Saxena I Pati S Singh S . Enhancing NADPH to restore redox homeostasis and lysosomal function in G6PD-deficient microglia. Heliyon. (2025) 11:e42735. doi: 10.1016/j.heliyon.2025.e42735 40084013 PMC11903804

[63] Stanton RC . Glucose-6-phosphate dehydrogenase, NADPH, and cell survival. IUBMB Life. (2012) 64:362–9. doi: 10.1002/iub.1017 22431005 PMC3325335

[64] Hontela A . Adrenal toxicology: environmental pollutants and the HPI axis. In: Mommsen TP Moon TW , editors. Biochem Mol Biol Fishes. IOS Press, Van Diemenstraat (2005). 6:331–63.

[65] Vander Boon J Van den Thillart GEEJM Addink ADF . The effects of cortisol administration on intermediary metabolism in teleost fish. Comp Biochem Physiol A. (1991) 100:47–53. doi: 10.1016/0300-9629(91)90182-c

[66] Secombes CJ Fletcher TC . The role of phagocytes in the protective mechanisms of fish. Annu Rev Fish Dis. (1992) 2:53–71. doi: 10.1016/0959-8030(92)90056-4

[67] Hardie LJ Ellis AE Secombes CJ . *In vitro* activation of rainbow trout macrophages stimulates inhibition of *Renibacterium salmoninarum* growth concomitant with augmented generationof respiratory burst products. Dis Aqua Organ. (1996) 25:175–83. doi: 10.3354/dao02517542402181

[68] Sharp GJE Secombes CJ . The role of reactive oxygen species in the killing of the bacterial fish pathogen *Aeromonas salmonicida* by rainbow trout macrophages. Fish Shellfish Immunol. (1993) 3:119–29. doi: 10.1006/fsim.1993.101321632986

[69] Manush SM Pal AK Das T Mukherjee SC . Dietary high protein and vitamin C mitigate stress due to chelate claw ablation in Macrobrachium rosenbergii males. Comp Biochem Physiol. (2005) 142:10–8. doi: 10.1016/j.cbpb.2005.07.004 16109496

[70] Zhang X Yang S Chen J Su Z . Unraveling the regulation of hepatic gluconeogenesis. Front Endocrinol (Lausanne). (2019) 9:802. doi: 10.3389/fendo.2018.00802 30733709 PMC6353800

[71] Bantidos MG Stalikas N Fyntanidou B Skalidis I Arvanitaki A Kofos C . The role of stress-induced hyperglycemia in acute coronary syndromes: pathophysiology, novel markers and clinical implications. Expert Rev Cardiovasc Ther. (2025) 23:723–42. doi: 10.1080/14779072.2025.2561068 40944560

[72] Ali D Nagpure NS Kumar S Kumar R Kushwaha B Lakra WS . Assessment of genotoxic and mutagenic effects of chlorpyrifos in freshwater fish Channa punctatus (Bloch) using micronucleus assay and alkaline single-cell gel electrophoresis. Food Chem Toxicol. (2009) 47:650–6. doi: 10.1016/j.fct.2008.12.021 19141310

[73] Abhay SY Bhatnagar A Kaur M . Aberrations in the chromosomes of *Cirrhinus mrigala* (Hamilton) upon exposure to butachlor. IJT. (2013) 7:860.

[74] Berdowska A Bandurska K . Health hazards associated with exposure to endosulfan: a mini-review. Toxics. (2025) 13:455. doi: 10.3390/toxics13060455 40559928 PMC12196845

[75] Kumar N Priyadarshi H Parhi J Pandey PK Kumar D . Acute toxicity of mercury in response to metallothionein expression and oxidative and cellular metabolic stress in Barbonymus gonionotus. Sci Rep. (2025) 15:12022. doi: 10.1038/s41598-025-95697-1 40199902 PMC11978849

[76] Da Cuna RH Vazquez GR Piol MN Guerrero NV Maggese MC Lo Nostro FL . Assessment of the acute toxicity of the organochlorine pesticide endosulfan in *Cichlasoma dimerus* (Teleostei, Perciformes). Ecot Environ Saf. (2011) 74:1065–73. doi: 10.1016/j.ecoenv.2011.02.002 21377734

[77] Nowak B . Histological changes in gills induced by residues of endosulfan. Aqua Toxicol. (1992) 23:65–84. doi: 10.1016/0166-445x(92)90012-c

[78] Evans DH Piermarini PM Choe KP . The multifunctional fish gill: dominant site of gas exchange, osmoregulation, acid-base regulation, and excretion of nitrogenous waste. Physiol Rev. (2005) 85:97–177. 15618479 10.1152/physrev.00050.2003

[79] Banaee M . Physiological dysfunction in fish after insecticides exposure. In: Trdan S , editor. Insecticides - Development of Safer and More Effective Technologies. InTech. (2013) p. 103–42.

[80] Henry RP Lucu C Onken H Weihrauch D . Multiple functions of the crustacean gill: osmotic/ionic regulation, acid-base balance, ammonia excretion, and bioaccumulation of toxic metals. Front Physiol. (2012) 3:431. doi: 10.3389/fphys.2012.00431 23162474 PMC3498741

[81] Ortiz JB De Canales MLG Sarasquete C . Histopathological changes induced by lindane (γ-HCH) in various organs of fishes. Sci Mar. (2003) 67:53–61. doi: 10.3989/scimar.2003.67n153

[82] Gill TS Pande J Tewari H . Hepatopathotoxicity of three pesticides in a freshwater fish, *puntius conchonius* ham. J Environ Sci Health A. (1990) 25:653–63. doi: 10.1080/1093452900937558737339054

[83] Altinok I Capkin E . Histopathology of rainbow trout exposed to sublethal concentrations of methiocarb or endosulfan. Toxicol Pathol. (2007) 35:405–10. doi: 10.1080/01926230701230353 17455089

